# Valedictory Address to the Graduating Class of the Session of 1854-5, in the Medical Department of the University of Buffalo

**Published:** 1855-03

**Authors:** Sanford B. Hunt

**Affiliations:** Professor of Anatomy


### BUFFALO MEDICAL JOURNAL

AND

### MONTHLY REVIEW.

**VOL. 10.** MARCH, 1855. **NO. 10.**

### ORIGINAL COMMUNICATIONS.

ART. I.—*Valedictory Address to the Graduating Class of the Session of 1854–5, in the Medical Department of the University of Buffalo.* By SANFORD B. HUNT, M. D., Professor of Anatomy. Published by request of the Class.

GENTLEMEN GRADUATES,

The period of preparation is completed. You occupy now that honorable position for which you have given three years of toil. During the passage of those years, the eager chase for gain, the fierce struggles of ambition, the noisy clashings of conflicting interests, have come to your ears like the sounds of Ocean to him who sits in quiet contemplation on the shore. But the time of your seclusion is over—you must pass from out the quiet haven of student-life to that rough sea, where the individual skill of each must be his protection from the storm. At your outset, I have been chosen by my colleagues to say the word of parting, and to bid you a hearty God-speed upon your voyage.

Two subjects form the usual burden of addresses like the present: first, the deep wound inflicted on the sensibilities by the melancholy parting between teacher and pupil; and following that, a Jeremiad on the retrograde tendencies of the age, the lost honors of medicine, and the unhappy proclivity of these latter days toward that medical bugbear, Quackery.

Do not imagine that I shall inaugurate a new era in valedictories by departing from these time-honored customs. It is the favorite sin of the medical mind to cling affectionately to bygones. But the few brief minutes allotted to me must prevent that full justice which I should like to render to some sacred subjects in valedictory lore, and confine me entirely to the two main points already announced. Should I, then, omit to pay due homage to the memory of Hippocrates, it is not that I willingly do so, but that my Grecian mythology is forgotten. Should I neglect to recount the vast debt owed by humanity to medicine, it is because I fear it may never be collected.

Our intercourse, gentlemen, has been pleasant. Nothing has occurred to make it otherwise, and we trust that you leave your Alma Mater with honest feelings of interest in her prosperity, and a determination to do honor to yourselves by honoring her. But to say that either you, or we, regret our coming separation would be an excess of sentiment. You have attained a long-sought honor—you are eager to go out to the world, and assert the strength that you have husbanded. *We* have pleasantly closed a winter of continuous labor and exertion for your interests. Bear with you *that* reflection, gentlemen, and it is needless for me to grow lachrymose to-night. While we feel that the intercourse of college-life has begotten many generous friendships among us all, and many glowing memories to cheer the thoughtful hours of future life, we would not have you go out from us with a puling sentimentality, with sickly reflections on the past, and cowardly fear of what is yet to come; but rather would we have you start gaily out upon a prosperous voyage, with a cheer upon your lips, an eye steadily fixed on the difficulties to be met, and a heart so brave and sound that fear can have no entrance.

Undoubtedly this is a moment for serious reflection. With the accession of new honors you have also assumed new responsibilities. The halcyon days are done. Before you are duties, cares, disappointments, and successes. All those hopes and failures which make up the sum of existence, are as inevitable to you as to other men—in the spirit with which you meet them lies the secret of your happiness.

It is but a poor encouragement to such a spirit, to be told that you are entering upon a profession of unusual difficulty, an occupation once dignified and cherished, now tottering to decrepitude, its feeble existence harassed by the hordes of quackery, and sustaining itself only by the aid of the few conservative prejudices, which have come down to this degenerate age from those good old times, when men were not human, and original depravity had not wrought its perfect work. This is said to be an age of humbug; but the veriest humbug begotten in its womb is that disgraceful cant, which deforms our medical literature, and utters its ululations in our medical oratory, declaring, with a vehemence worthy the magnitude of the lie, that legitimate medicine is incapable of sustaining itself against this fabulous outside pressure of the age.

Gentlemen, if medicine is really in opposition to the spirit of the age, if it foolishly clings to antiquated doctrines and the dogmata of scholiasts, to the exclusion of a wholesome tendency to improvement, you have committed an egregious blunder in seeking an entrance to its ranks. But lest we decide this question too hastily, let us look at the real status of the profession at the present time.

No institution on earth presents such a catholic uniformity of sentiment, or is so widely scattered in every land. Medical men from every town where civilization exists, throughout the wide world, might meet in Congress with common interests, a common code of ethics, and a common literature. Such a Congress would represent all the various interests of learning; for it is a fact of glorious significance, that natural science and the useful arts owe half their vigor to the discursive researches of medical men. A large proportion, probably more than half the common stock of human learning is found within the pale of our profession.

Is it possible that such an organization can be defeated by any pressure from without? Never, gentlemen, so long as its present catholic tendencies remain; but whenever we open our ranks to one-idea-ism, whenever we link ourselves to any special doctrines as represented by homœopathy, or what is ridiculously called eclecticism, the ægis of our safety is destroyed!

And this brings me to the assertion of what constitutes the distinction between legitimate medicine and quackery. Our science is not made up of doctrines—it is only a mass of facts arranged with careful accuracy, and he who best interprets them is the best physician. A brief history of the curriculum of study pursued in this country, and in Europe, will best express its fundamental principles.

The tyro, on presenting himself as a student, is examined with reference to his proficiency in those ordinary branches of learning, without which it is as impossible to be a physician, as to be a gentleman. This proving satisfactory, he begins a course of study. Separate professorships are employed in teaching him anatomy, physiology, pathology, materia medica, and chemistry. Thus these several branches are taught by individuals whose studies have been directed to their special subjects.

Doctrine as such — the peculiarities of legitimate medicine — have here no place. The student simply acquires familiarity with the arrangements, functions, and diseases of the different organs; with drugs, their properties and actions; with remedies as distinguished from drugs in the forms of air, sunshine, water, diet and exercise; and with the chemical reäctions of the organs upon the blood, which so nearly approach the solution of the great problem — Life.

So far the student has learned only facts. These are simply demonstrative sciences. All minds receive their facts alike, and accord to them but one interpretation. These collateral studies complete, it is the province of others to teach their application to the treatment of disease, and to still further apply them to those maladies and dangers entailed by Mother Eve upon her sex.

How, then, is practical medicine inculcated? Simply by a history of facts. The student is told the remedies which experience has sanctioned, the rule of safety in their administration, and especially the fallacy of trusting too much to medication.

He is, moreover, exhorted to bind himself to no Procrustean system, to keep his mind open to suggestions of improvement, and, looking upon medicine as an imperfect art, to devote his energies to its amendment.

Thus by a somewhat tedious route we have reached the distinction which separates us from empiricism. Our votaries are taught to seek their remedies wherever they may find them. All the treasures of the earth, the air, and sea are ours. The multitudinous forms of vegetable life afford their juices and their cunning extracts; from the dark caves of earth the chemist robs the subtle mineral, tortures it by fire, or melts it in his greedy acids, for our use. From the very grottoes of the mermaid the fish is captured, whose oil shall bring fatness and health to the wasted consumptive; while from that waif of ocean, the sea-weed, for centuries the very type of worthlessness, the *inutile alga* of Horace, is brought the precious iodine. Air, food, and sunlight yield their tribute, while the universal water stills its babblings to assist our art. And over all presides the calm spirit of the physician, whose quiet prophecy of help and hope aids in its own fulfillment.

Contrast this with the narrow spirit of sectional medicine. Bound down by an immitigable creed, the homœopath loses the precious moment when one heroic drug might change the trembling chances, and bring the color to the cheek again. The slave of a prodigious over-estimate of the powers of water, the hydropath neglects those simpler remedies to whose worth the ages testify. And the eclectic, imprisoned in the narrow limits of vegetable power, allows the mineral kingdom, the right arm of therapeutics, to lie in useless idleness. With them is slavery to creeds — with us is liberty, freedom to select whatever remedy God places in our hands.

It is this universality of legitimate medicine that begets the varied forms of quackery. To the weak or ignorant mind a creed is a necessity: it leans upon it for protection from its own imbecility; or in the case of that form of intellect which loves the marvelous, and is given over to an excess of refinement, it seeks its analogue in medicine, and finds in the subtle nothingness of Hahnemahan, the twin-sister to its own spiritual tendencies. It is a curious feature in this phase of mental philosophy, that innate differences of intellect, the comparative strength of the reasoning, perceptive, or imaginative faculties, govern their owner in the choice of a medical system. It has been said that seven-eighths of the followers of Swedenborg are also devotees of Hahnemahn, and all must have noticed a similar proclivity in the spiritualists of the present day. It is, at any rate, sufficiently evident that medicine is not exempt from the influences which govern systems of belief. An important inference may be reached from this view of the question, viz: that legitimate medicine is unsuited to the peculiarities of certain minds, and will never obtain their confidence. A thorough education of the people would doubtless somewhat modify this result; but education only directs, does not mould, the mind. At the first opportunity it will slip off the shackles of the schools, and assert its original tendencies.

Quackery, then, is immortal. Changing its forms to suit the special fantasy of the day, it will ever adapt itself to superstition, ignorance, or super-refinement. As these three elements make up a large proportion of living humanity, it is equally evident that they must cause a considerable deduction from the honors and profits of the regular profession.

When all churches shall have merged in one; when the imaginative Swedenborgian is satisfied with cool Presbyterian logic; when the gross and sensual Mormon can adopt the calm, undemonstrative religion of the Episcopalian; when all varieties of dissent shall give up that independence for which so many thousands have suffered exile, imprisonment, and death, to submit their consciences and creeds to the keeping of the Papacy; when Romanism itself shall abandon its altars to an elective clergy; then, gentlemen, and not before, shall we have the countenance and confidence of the entire people.

But this is not the only inference. The larger mass of community are sober-minded, and to some degree conservative. The very qualities of empiricism which attract erractic minds, are sure to repel the thoughtful and considerate. Hence it is, that in the perpetual strife of medical opinions, the regular profession has ever stood as the representative of medicine; recognized as such alike by the tenor of our laws, and an overwhelming public sentiment. The public mind acknowledges that true Eclecticism which is the characteristic trait of legitimacy, and he who deserts his profession for the practice of quackery inevitably loses social position and caste. We never hear of reputations won in quackery — always of the money made — and yet, if we shut out of view the few great fortunes made by inventors of omnipotent pills, we shall see that even this is most often an *ignis fatuus*, which dazzles, to betray its followers into the very mire of poverty. The strong argument of money is in our favor.

Such desertions are extremely uncommon, and never occur among men of acknowledged excellence. Homœopathy, more than any other form of error, has detracted from our numbers, but not at all from our character. The cunning money-maker, or the disappointed and ill-employed, men who have waded in doubt for years together, mistaking their own incompetence for the faults of a system, have furnished the only proselytes.

There is an eccentric force in our profession which, like the rapid revolutions of a wheel, casts off the dirt which clings to its circumference. There is a rapidity of progress, a fierce and grasping energy, a relentless competition, which should warn the feeble-minded to stand back. None but the keen, quick, appreciative mind is fit for us. The constant accessions to medical literature and knowledge, the varied accomplishments which year by year are becoming more and more necessary to the physician, bewilder and distress the slow and incompetent. The effect of all this rush and vigor is like that panic we sometimes see in railroad depots, when some good, quiet soul, startled from his propriety by the scream of the whistle and the noisy outcries of the crowd, falls in great distress of mind about his baggage, and finally rushes insanely off at fifty miles per hour — and that in the wrong train.

"Nothing gives a sadder sense of decay than this loss or suspension of the power to deal with unaccustomed things, and to keep up with the swiftness of the passing moment. It can merely be a suspended animation; for, were the power actually to perish, there would be little use of immortality. We are less than ghosts, for the time being, whenever this calamity befalls us.*"

It is then with a sense of infinite pity that we behold these occasional desertions. It is not so much an error of the heart, as a feeble imbecility, a nerveless, forceless incapacity to maintain a position of equality.

---

* Hawthorne's "House of the Seven Gables."

But, gentlemen, do all of those who adhere faithfully to our communion live up to these requirements of untiring energy and labor? Are they all men of the age, men of vigor, strength, and study? Alas! all clergymen are not divines, all lawyers are not jurists, all M. D.'s are not doctors. It is a rare compound of tact and study, of knowledge both of books and men, that makes the truly competent physician. We cannot claim such excellencies for all our brethren, and can only say, and that with sad and mournful truth, that the public get all the medical talent they pay for!

Our standard of excellence is not established by any general average of talent, but by the attainments of those more brilliant minds who necessarily form the minority, or rather the leaders of the profession. It is barely possible that all men may have been created equal; but if they were, they have grown up with a marvelous departure from the original design. It cannot be expected that men of intellectual power, possessed of the consciousness that the thoughts of their brains have an actual cash value, will flock in any numbers to a profession which does not pay as well as the mechanic arts. One is at a loss, here, whether to assign the fault to the public or to ourselves.

Useful talent is pretty sure to bring a fair price except in an overcrowded market. There would be some assumption in assuming that the market has been overstocked with available medical skill. Any such claim would meet with a very emphatic denial, and we can only assert that the profession of medicine performs so large an amount of gratuitous labor, as to shut out from its practice a great number of intelligent and useful men, who believe that charity begins at home, and are not at all disposed to accept of Irish blessings in exchange for learned opinions.

Your speaker feels some hesitation in intruding so commonplace a matter as dollars and cents before an audience so æsthetic; but however much we may admire the true and beautiful, however loftily the soul may look down upon the filthy lucre, the hand is ever open for its reception.

That is a very generous and romantic sentiment which makes a Lady Bountiful of the profession, and exhorts its members to do everything in charity for the love of God; but its justice may be questioned. Our state Code of Ethics quotes old Boerhaave, as saying that "The poor are the best customers, because God will be the paymaster." From now till doomsday is a long credit, unless we take the computation of our Second Advent friends. All this generous sentiment, so lavishly used in recounting the duties of the physician to the poor, will bear in the light of Justice but one interpretation: that a humane profession, thrown daily into contact with the sufferings of poverty, will find a thousand opportunities for unobtrusive charity, which the instinctive sympathies of a common humanity will dictate and employ; but it does not, cannot, mean that we of all the world should neglect the law of self-preservation; it cannot be intended that the sacred duty of charity, so imperative upon the rich, should be entirely foisted off to the care of the penniless physician.

The most enthusiastic student can be startled from the charm of books by the rough entrance of a dun, and many a glorious spirit has found his eager ambition for distinction and usefulness drowned in the bitter waters of poverty. Could the private history, the secret thoughts, the gnawing care which eats out the heart, chokes the ambition, and benumbs the spirit of the young physician, as he struggles through those long years of waiting of his early professional life, hoping, toiling, praying, cursing, the apples of Hesperides hung just beyond his outstretched arm, the waters of hope bubbling up to his very lip, but still untasted, conscious of strength, but bound by the green withes of a hated youthfulness — could all this history of aching hearts be known, there would be less admiration of that fatal sentimentalism which has made a great charity of the medical profession, and thrust upon the feeble shoulders of its youthful members the whole support of the sickness of the poor.

This is no extravagant statement; its verity is testified, not only by the general voice of the profession, but by the letter of our poor-laws. All over our land are great hospitals endowed by the state, the tax on the property of the physician contributing to their support. In the management of these hospitals every man, from the turtle-fed Governor down to the most forlorn of orderlies, is compensated for his services with the single exception of the physician. He alone, for some fancied advantage in reputation or experience, is condemned, unpaid, to a service always revolting, and often dangerous.

And so in every department of our public charities. All our prisons and almshouses are served by medical men at a price, which has grown so disgracefully small that the constituted medical authorities have declared it dishonorable to accept such appointments, and have boldly placed the question in such a shape that only the chiffoniers of the profession can serve under public pay.

Look to the field of private practice. During the past year — one of pestilence among the poor — the medical men of this city have given at least $50,000 in gratuitous services to the needy. This is too low an estimate, but if we compare even it with the actual income of the profession here, we shall find that it has lent to the Lord not one-tenth, but rather one-third of the fruits of the year. There is a manifest injustice in this which needs no comment, but it has other bearings and influences which interest the public rather than ourselves. The tendency of this system is to discourage and dishearten the entrance of talent to the profession, and thus, by securing an inferior order of medical men, to reäct disastrously upon the public. I have spoken thus freely of this evil, because I believe it to be an insurmountable barrier to medical reform; and further, because the conduct of any scheme for its removal must rest upon the young men of the profession.

But do not mistake the principles which should govern it. Charity has ever been the brightest of medical virtues, and the devotion of the physician to the poor has given him his strongest hold upon the sympathies and affection of the public. To refuse relief to the cry of God's image in distress, would involve an injustice deeper than your own. The individual pauper is a fellow-man, suffering from social laws for which he is not responsible. Do not add to the sense of wrong, forever dimly burning in his heart, by withholding your aid in the hour of sickness. But the *public* pauper is a ward of the great body-politic—let his guardians protect him!

No hour, gentlemen, in the history of American medicine has presented such auspicious omens as mark your commencing career. The profession is strong. In spite of the loss of our civil privileges, and of the thousand adverse surroundings incident to professional progress in a new and shifting country, that *esprit du corps* which has never left us, has secured a glorious fruitage of added learning and means of usefulness. The best man of fifty years ago was hardly a match for the tyro of to-day. Our literature is rich and thrifty beyond all comparison, and has made a philosophical science of that which was once an uncertain art. A new want, an unheard-of necessity, threatens the people; the want of medical men, not only men of genius and study, but men, such as can be got, good, bad, or indifferent, to fill the growing hiatus in the ranks of the profession. For the last five years every season has witnessed a falling off in the number of aspirants for medical honors. California, and the mighty impulse it gave to commercial activity, has opened a thousand rapid avenues to wealth, and absorbed the youthful energy and vigor of the country. The colleges and the public are the losers; the profession gains immensely. Over-crowded beyond all endurance a little while ago, its ranks are now open, and vacancies seek applicants, not applicants vacancies. The rural districts are already calling for more men, but the end is not yet. The present order of things must continue for awhile, and the profession will soon, gathering strength from the very smallness of its numbers, stand like a monopolist in the market, and make its own conditions.

At such a moment, gentlemen, you leave us to try your strength in the rough battle of the world. In this parting address I have spoken of your duty to yourselves and to your profession. I have urged upon you a firm, unyielding policy, which looks rather to its own strength, than to the justice of the world for its success. Follow it out manfully, and you shall win; but forget not that you owe, also, duties to the public. Work! There is no success in life without labor. God has so formed us, that our cheerful compliance with the Adamic curse deprives it of half its punishment. Work in this sense is a luxury; and he who sits with folded hands, watching the busy life around, will one day find himself alone, forgotten, uncared-for, the slave of his own inactive Will.

Let me again protect myself from misapprehension. Fulfill with patience those duties a false idea of charity imposes upon you; but work like men to place those duties where they belong. Our history is full of bright examples of courageous charity which it will be your duty to emulate. It is but a little while ago—the actors in the scene are still young—that twelve young men entered, unpaid, as assistants in the hospital at Bellevue. At the end of the year the sod was yet fresh upon the undistinguished graves of seven of that little band, and none but the families thus made desolate knew that heroes had perished. The mother's wail was as loud, the father's heart wrung by as keen a grief as that which, at that very moment, a nation offered as the requiem of soldiers fallen on the battle-fields of Mexico, their faces to the foe. That calmer, deeper, and more Godlike bravery which faced the pestilence at Bellevue, found not even a record in the daily prints. No pageant, no banners of woe, no mausoleum, witnessed their interment; and here, to-night, I cannot tell their names, and know the facts only from their casual mention by one of the five survivors, your teacher of Practice.

In time of epidemics the physician assumes his real and noblest character. No longer mindful of his own dangers or necessities, he is found wherever the shadow of the wing of Azrael is darkest, wherever the wail of his victim is heard. In such an hour, in sleeplessness, fatigue and danger, He who loveth the poor gives him strength and courage, and his calm countenance, lighted by hope and confidence, brings health and joy to the bedside deserted by all others.

Gentlemen, you are impatient. No longer our scholars, but our equals, you long for the last word of the last lecture, that you may go fearlessly forth to push your way, trusting to clear heads, youthful energies, and firm resolve. They will not fail you. I have told you that the profession is strong, strong to assert its rights, strong to maintain them, strong against quackery, and strongest in its hallowed mission. You are our offering for its sustenance. There is work for all. Give us your strength to build and perfect the temple of our art.

"See where aloft its hoary forehead rears,
The towering pride of twice a thousand years!
Far, far below the vast incumbent pile,
Sleeps the broad rock from Art's Ægean isle;
Its massive courses, circling as they rise,
Swell from the waves, and mingle with the skies;
There every quarry lends its marble spoil,
And clustering ages blend their common toil;
The Greek, the Roman, reared its mighty walls;
The silent Arab arched its mystic halls;
In that fair niche, by countless billows laved,
Trace the deep lines that Sydenham engraved;
On yon broad front, that breasts the changing swell,
Mark where the ponderous sledge of Hunter fell;
By that square buttress, look, where Louis stands,
The stone yet warm from his uplifted hands."

